# The zinc cluster transcription factor Znc1 regulates Rta3-dependent miltefosine resistance in *Candida albicans*

**DOI:** 10.1128/msphere.00270-24

**Published:** 2024-06-11

**Authors:** Bernardo Ramírez-Zavala, Ines Krüger, Sonja Schwanfelder, Katherine S. Barker, P. David Rogers, Joachim Morschhäuser

**Affiliations:** 1 Institute of Molecular Infection Biology, University of Würzburg, Würzburg, Germany; 2 Department of Pharmacy and Pharmaceutical Sciences, St. Jude Children’s Research Hospital, Memphis, Tennessee, USA; University of Georgia, Athens, Georgia, USA

**Keywords:** *Candida*, miltefosine, Tac1, Znc1, Rta3, drug resistance, gene regulation, transcription factor

## Abstract

Zinc cluster transcription factors (ZCFs) are a family of transcription regulators that are almost exclusively found in the fungal kingdom. Activating mutations in the ZCFs Mrr1, Tac1, and Upc2 frequently cause acquired resistance to the widely used antifungal drug fluconazole in the pathogenic yeast *Candida albicans*. Similar to a hyperactive Tac1, a constitutively active form of the ZCF Znc1 causes increased fluconazole resistance by upregulating the multidrug efflux pump-encoding gene *CDR1*. Hyperactive forms of both Tac1 and Znc1 also cause overexpression of *RTA3*, which encodes a seven-transmembrane receptor protein involved in the regulation of asymmetric lipid distribution in the plasma membrane. *RTA3* expression is also upregulated by miltefosine, an antiparasitic drug that is active against fungal pathogens and considered for treatment of invasive candidiasis, and *rta3*Δ mutants are hypersensitive to miltefosine. We found that activated forms of both Tac1 and Znc1 confer increased miltefosine resistance, which was dependent on *RTA3* whereas *CDR1* was dispensable. Intriguingly, the induction of *RTA3* expression by miltefosine depended on Znc1, but not Tac1, in contrast to the known Tac1-dependent *RTA3* upregulation by fluphenazine. In line with this observation, *znc1*Δ mutants were hypersensitive to miltefosine, whereas *tac1*Δ mutants showed wild-type tolerance. Forced expression of *RTA3* reverted the hypersensitivity of *znc1*Δ mutants, demonstrating that the hypersensitivity was caused by the inability of the mutants to upregulate *RTA3* in response to the drug. These findings establish Znc1 as a key regulator of miltefosine-induced *RTA3* expression that is important for wild-type miltefosine tolerance.

**IMPORTANCE:**

Transcription factors are central regulators of gene expression, and knowledge about which transcription factor regulates specific genes in response to a certain signal is important to understand the behavior of organisms. In the pathogenic yeast *Candida albicans*, the *RTA3* gene is required for wild-type tolerance of miltefosine, an antiparasitic drug that is considered for treatment of invasive candidiasis. Activated forms of the transcription factors Tac1 and Znc1 cause constitutive overexpression of *RTA3* and thereby increased miltefosine resistance, but only Tac1 mediates upregulation of *RTA3* in response to the known inducer fluphenazine. *RTA3* expression is also induced by miltefosine, and we found that this response depends on Znc1, whereas Tac1 is dispensable. Consequently, *znc1*Δ mutants were hypersensitive to miltefosine, whereas *tac1*Δ mutants showed wild-type tolerance. These findings demonstrate that Znc1 is the key regulator of *RTA3* expression in response to miltefosine that is important for wild-type miltefosine tolerance.

## INTRODUCTION

Zinc cluster transcription factors (ZCFs) are a family of transcription regulators that are almost exclusively found in the fungal kingdom (1). The pathogenic yeast *Candida albicans* possesses 82 genes encoding ZCFs, as defined by the signature motif CX_2_CX_6_CX_5-24_CX_2_CX_6-9_C in their DNA binding domain (2, 3). Several members of the ZCF family have attracted particular attention because they can mediate resistance to the widely used antifungal drug fluconazole, which inhibits ergosterol biosynthesis. Upc2 regulates the expression of ergosterol biosynthesis genes, and Tac1 and Mrr1 control the expression of *CDR1*/*CDR2* and *MDR1*, respectively, which encode multidrug efflux pumps (4–7). Gain-of-function (GOF) mutations in these transcription factors result in constitutive overexpression of their target genes and are a common cause of acquired fluconazole resistance in clinical *C. albicans* isolates (4, 6, 8–17).

ZCFs can also be artificially activated by fusing the heterologous Gal4 activation domain from *Saccharomyces cerevisiae* to their C-terminus, which in the case of Upc2, Tac1, and Mrr1 mirrored the effects of naturally occurring GOF mutations in these transcription factors (3). Screening of a library of *C. albicans* strains that expressed all *ZCF* genes in such a potentially activated form identified several additional transcription factors that conferred increased fluconazole resistance. One of these genes, *ZNC1*, is located directly upstream of *TAC1* on chromosome 5. Similar to activated forms of Tac1, the artificially activated Znc1 (hereafter referred to as Znc1*) caused overexpression of *CDR1* (but not *CDR2*), and the increased fluconazole resistance conferred by Znc1* was lost in *cdr1*Δ mutants (3).

Transcriptional profiling of strains expressing ZNC1* showed that one of the most highly upregulated genes (>80-fold by DNA microarray analysis) was *RTA3*, which is also a Tac1 target gene (3, 4, 18, 19), and Znc1 has recently been shown to bind to the *RTA3* promoter region (20). *RTA3* encodes a seven-transmembrane receptor protein involved in the regulation of asymmetric lipid distribution in the plasma membrane (21, 22). It is constitutively upregulated in fluconazole-resistant, clinical *C. albicans* isolates containing GOF mutations in Tac1, and its expression is also induced by the drug fluphenazine in a Tac1-dependent manner (4, 18, 19, 23, 24). While deletion of *RTA3* in a *C. albicans* isolate that overexpressed the gene due to a GOF mutation in Tac1 also resulted in slightly increased fluconazole-susceptibility (25), *RTA3* deletion in a derivative of the *C. albicans* reference strain SC5314 did not affect fluconazole sensitivity (22).

Interestingly, *RTA3* expression was recently found to be upregulated by miltefosine, an antiparasitic drug that is also active against fungal pathogens and considered for treatment of invasive candidiasis (https://www.accessdata.fda.gov/scripts/opdlisting/oopd/detailedIndex.cfm?cfgridkey=843921), and deletion of *RTA3* in strain SC5314 resulted in hypersensitivity to miltefosine (22). We therefore investigated whether miltefosine sensitivity is regulated by Znc1 and if hyperactivity of this transcription factor confers increased miltefosine resistance.

Note to readers: in this report, the words resistance, tolerance, sensitivity, etc. are used in their traditional sense and do not refer to growth above or below specific breakpoints.

## RESULTS

### A hyperactive form of Znc1 confers Rta3-dependent miltefosine resistance

**Fig 1:**
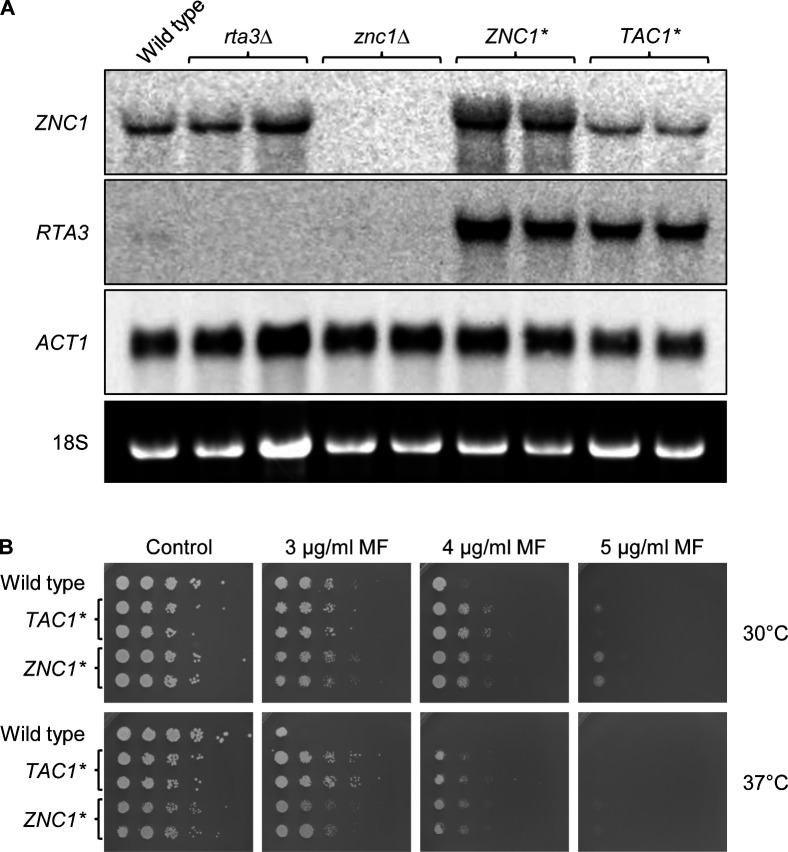
A hyperactive form of Znc1 causes constitutive *RTA3* overexpression and increased miltefosine resistance. (**A**) Detection of *ZNC1* and *RTA3* transcripts in the wild-type parental strain SC5314 and derivatives expressing ZNC1* or TAC1* from the *ADH1* promoter; *rta3*Δ and *znc1*Δ mutants served as negative controls. Yeast extract-peptone-dextrose (YPD) overnight cultures of the strains were diluted 10^−2^ in fresh YPD medium and grown for 4 h at 30°C. Total RNA was isolated and used for Northern hybridization with digoxigenin-labeled *ZNC1*, *RTA3*, and *ACT1* (control mRNA) probes. The identities of the transcripts are indicated on the left side of the blots; the 18S rRNA on the ethidium bromide-stained gel served as additional loading control. (**B**) Miltefosine sensitivity of the wild-type strain SC5314 and derivatives expressing the hyperactive ZNC1* or TAC1* alleles. Serial 10-fold dilutions of the strains were spotted on synthetic defined (SD) agar plates without (control) or with the indicated miltefosine (MF) concentrations and incubated for 2 days at 30°C or 37°C. Two independently constructed series of strains were used in panels **A** and **B**.

To confirm the upregulation of *RTA3* by Znc1*, which was previously observed using DNA microarrays, we compared *RTA3* expression in the wild-type parental strain SC5314 and derivatives containing hyperactive ZNC1* and TAC1* alleles by Northern hybridization. Figure 1A shows that *RTA3* mRNA levels, which were barely detectable in the wild type, were strongly upregulated by both Znc1* and Tac1*. To test if the overexpression of *RTA3* caused by Znc1* and Tac1* resulted in increased resistance to miltefosine, drug sensitivity assays were performed on plates containing different miltefosine concentrations. As seen in Fig. 1B, both hyperactive transcription factors conferred increased miltefosine resistance. Growth of the wild-type strain SC5314 was more strongly inhibited by miltefosine at 37°C compared with 30°C, and at a miltefosine concentration of 3 µg/mL, increased resistance of the strains containing ZNC1* or TAC1* was observed when the strains were grown at 37°C but not when incubated at 30°C. At this miltefosine concentration, TAC1* enabled better growth than ZNC1*, which was due to ZNC1* causing a stronger fitness defect than TAC1* in the absence of the drug, visible by the reduced growth of these strains on the control plates. On plates containing 4 µg/mL miltefosine, increased drug resistance conferred by ZNC1* and TAC1* was observed at both temperatures. At 5 µg/mL miltefosine abolished growth at 37°C of all strains.

**Fig 2:**
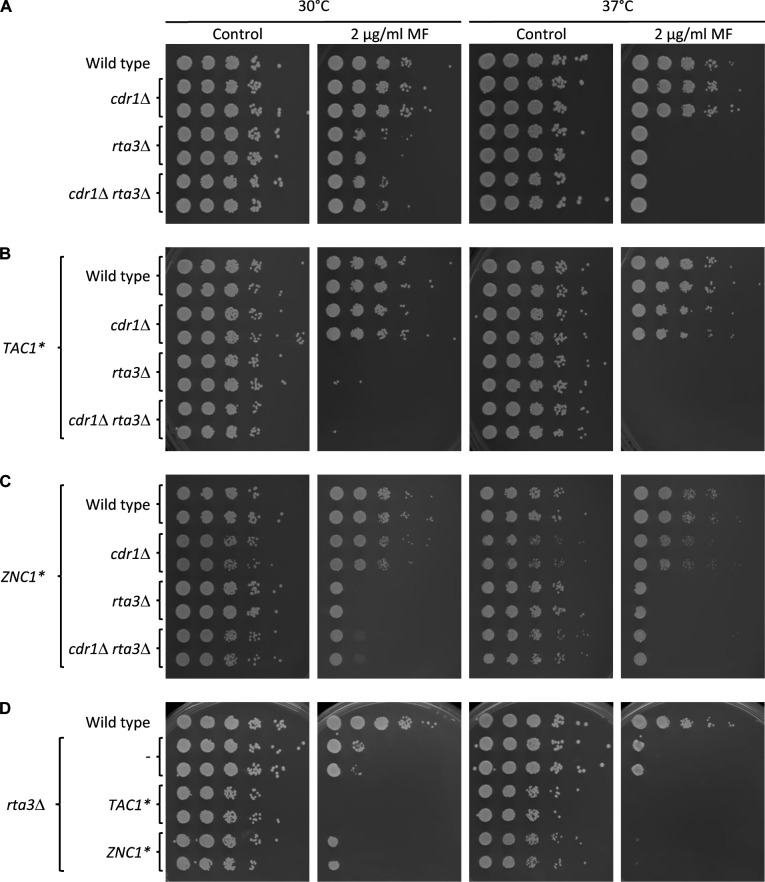
Znc1-mediated miltefosine resistance depends on Rta3. Serial ten-fold dilutions of the wild-type strain SC5314, *cdr1*Δ and *rta3*Δ single mutants, *cdr1*Δ *rta3*Δ double mutants, and derivatives expressing the hyperactive ZNC1* or TAC1* alleles were spotted on SD agar plates without (control) or with 2 µg/mL miltefosine (MF) and incubated for 2 days at 30°C or 37°C. Two independently constructed series of strains were used.

We then tested if the increased miltefosine resistance conferred by the hyperactive transcription factors was caused by the overexpression of *RTA3* or other Znc1 and Tac1 target genes. Deletion of *RTA3* in the wild-type strain SC5314 resulted in increased miltefosine susceptibility (Fig. 2A), confirming previous findings (22). In contrast, deletion of *CDR1*, which encodes the major *C. albicans* drug efflux pump and is a target of both Znc1 and Tac1, did not increase miltefosine susceptibility in the presence or absence of *RTA3* (Fig. 2A). The increased miltefosine resistance conferred by Znc1* and Tac1* in a wild-type background was completely abolished in the absence of *RTA3* (Fig. 2B and C). In fact, in the absence of *RTA3*, strains containing the hyperactive transcription factors were even more sensitive to miltefosine than *rta3*Δ mutants, as is evident in the direct comparison of the strains on the same plates (Fig. 2D). These results demonstrate that the strong *RTA3* overexpression causes the increased miltefosine resistance of strains with hyperactive forms of Znc1 and Tac1, which would otherwise even become hypersusceptible to the drug.

### Znc1 is required for miltefosine-induced *RTA3* expression and wild-type miltefosine tolerance

**Fig 3:**
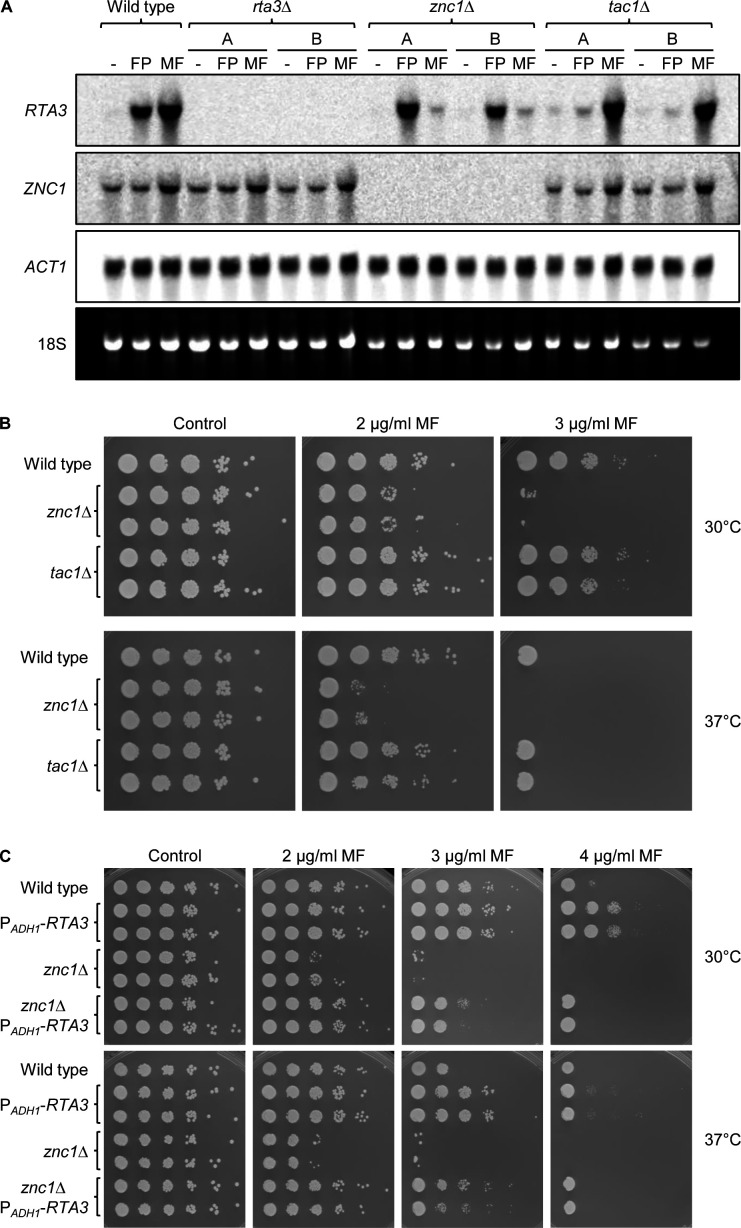
Znc1 mediates miltefosine-induced *RTA3* expression. (**A**) Detection of *RTA3* and *ZNC1* transcripts in the wild-type parental strain SC5314 and *znc1*Δ and *tac1*Δ mutants; *rta3*Δ mutants served as negative controls. Log-phase cultures of the strains were grown for 1 h in the absence or presence of 10 µg/mL fluphenazine (FP) or 10 µg/mL miltefosine (MF) and analyzed by Northern hybridization with *RTA3-*, *ZNC1-*, and *ACT1*-specific probes. The identities of the transcripts are indicated on the left side of the blots; the 18S rRNA on the ethidium bromide-stained gel served as additional loading control. (**B and C**) Miltefosine sensitivity of the indicated strains in a dilution spot assay on SD agar plates without (control) or with the specified miltefosine (MF) concentrations. The plates were incubated for 2 days at 30°C or 37°C. Two independently constructed series of mutants were used in (**A**) to (**C**).

*RTA3* expression has been shown to be induced by miltefosine in addition to previously known inducing drugs, such as fluphenazine (22). We therefore tested if Znc1 also has a role in miltefosine-induced *RTA3* upregulation. As seen in Fig. 3A, treatment with either 10 µg/mL fluphenazine or 10 µg/mL miltefosine resulted in strongly elevated *RTA3* mRNA levels in wild-type cells (the weak signal in the absence of an inducer did not allow a reliable quantification). *RTA3* induction by fluphenazine was dependent on Tac1 (mRNA levels were six-fold lower in *tac1*Δ mutants than in the wild type), but not on Znc1 (117% of wild-type levels in *znc1*Δ mutants), in line with findings by other researchers (20). Conversely, miltefosine-induced *RTA3* expression was strongly reduced in *znc1*Δ mutants (8% of wild-type levels), but occurred normally in the absence of Tac1 (101% of wild-type levels). The latter result is in contrast to that of a previous study, in which the induction of *RTA3* by miltefosine was reported to completely depend on Tac1 (22) (see also Discussion). The remaining weak induction of *RTA3* by miltefosine in the *znc1*Δ mutants might be mediated by Tac1.

**Fig 4:**
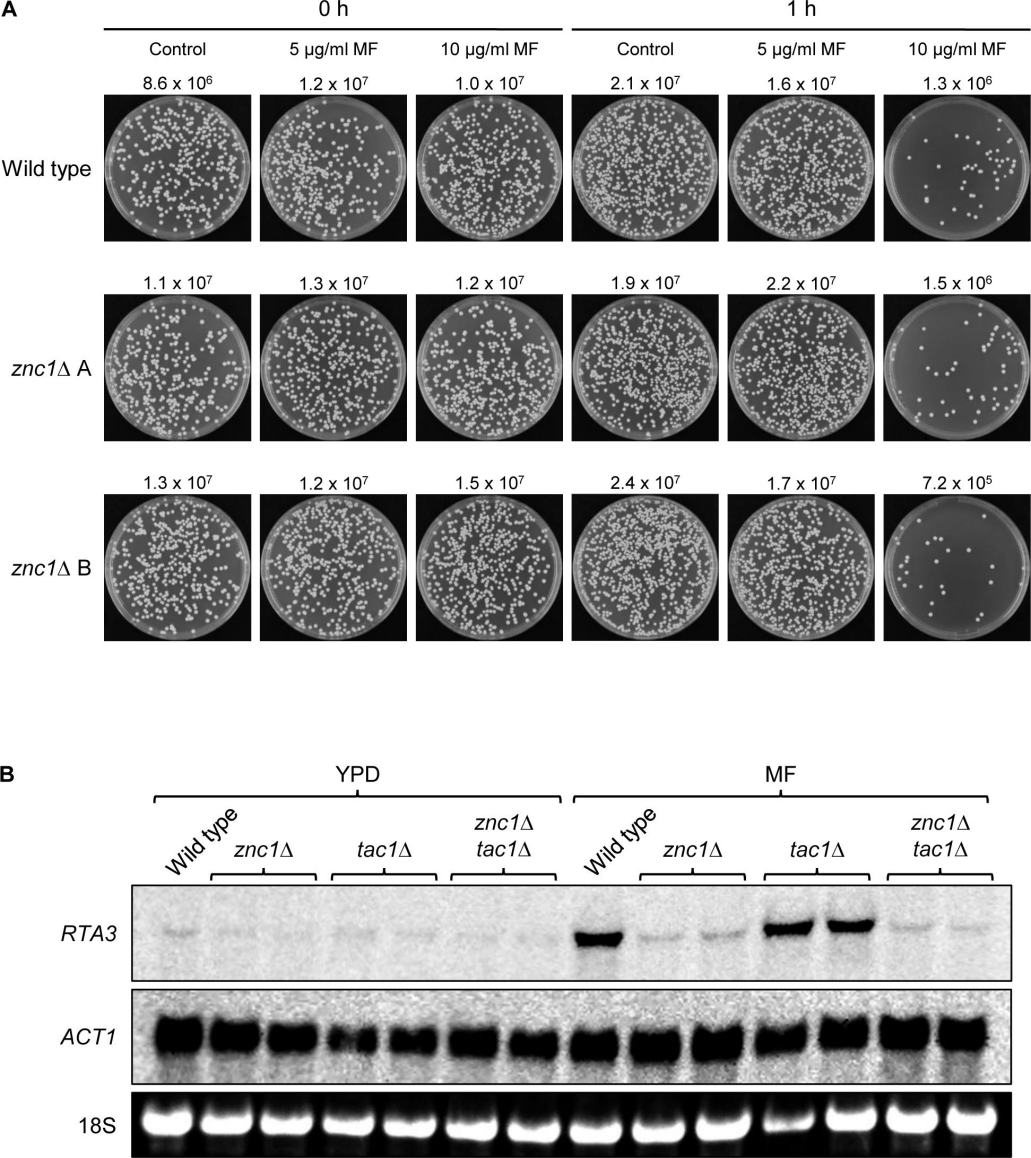
Effect of miltefosine on cell viability and *RTA3* expression. (**A**) YPD log-phase cultures of the wild-type parental strain SC5314 and the two independently generated *znc1*Δ mutants were grown for 1 h in the absence (control) or presence of the indicated concentrations of miltefosine (MF). Samples were taken at time point 0 (just after the addition of miltefosine) and after 1 h of further incubation at 30°C. Appropriate dilutions were spread on YPD plates and incubated for 2 days at 30°C to determine the number of viable cells. CFU counts are the average from two biological replicates, and one representative plate is shown in each case. (**B**) YPD log-phase cultures of the wild-type parental strain SC5314, *znc1*Δ and *tac1*Δ single mutants, and *znc1*Δ *tac1*Δ double mutants were grown for 30 min in the absence (−) or presence of 5 µg/mL miltefosine (MF) and analyzed by Northern hybridization with *RTA3-* and *ACT1*-specific probes. The identities of the transcripts are indicated on the left side of the blots. The 18S rRNA on the ethidium bromide-stained gel served as additional loading control. Two independently constructed series of mutants were used.

Because *RTA3* overexpression resulted in increased miltefosine resistance and *RTA3* induction by miltefosine strongly depended on Znc1, we wondered if Znc1 is required for wild-type miltefosine tolerance and compared the miltefosine sensitivity of the wild type and *znc1*Δ and *tac1*Δ mutants. As seen in Fig. 3B, *znc1*Δ mutants were hypersensitive to miltefosine, whereas *tac1*Δ mutants behaved like the wild type. Furthermore, expression of an additional *RTA3* copy from the *ADH1* promoter reverted the miltefosine hypersusceptibility of the *znc1*Δ mutants (Fig. 3C), as did reintroduction of *ZNC1* under control of the same promoter (Fig. S1). Because the *znc1*Δ mutants were hypersensitive to miltefosine, we wondered if their failure to induce *RTA3* expression was caused by cell killing at the miltefosine concentration used for the Northern hybridization experiment. We therefore tested the viability of cells grown for 1 h in the presence of different miltefosine concentrations. Treatment of YPD cultures with 10 µg/mL miltefosine reduced the number of viable cells, without a difference between the wild type and *znc1*Δ mutants, whereas treatment with 5 µg/mL miltefosine did not affect viability (Fig. 4A). We therefore tested *RTA3* induction by miltefosine in cells treated for only 30 min with 5 µg/mL miltefosine. We also constructed *znc1*Δ *tac1*Δ double mutants that were included in the experiment. As seen in Fig. 4B, *RTA3* expression was induced by miltefosine also under these conditions in the wild type and *tac1*Δ mutants, and the induction was abolished in *znc1*Δ and *znc1*Δ *tac1*Δ mutants. Collectively, these results demonstrate that upregulation of *RTA3*, mediated by Znc1, is important for the ability of *C. albicans* to withstand and grow in the presence of subinhibitory miltefosine concentrations.

### Znc1 is required for miltefosine tolerance in other clinical *C. albicans* isolates

**Fig 5:**
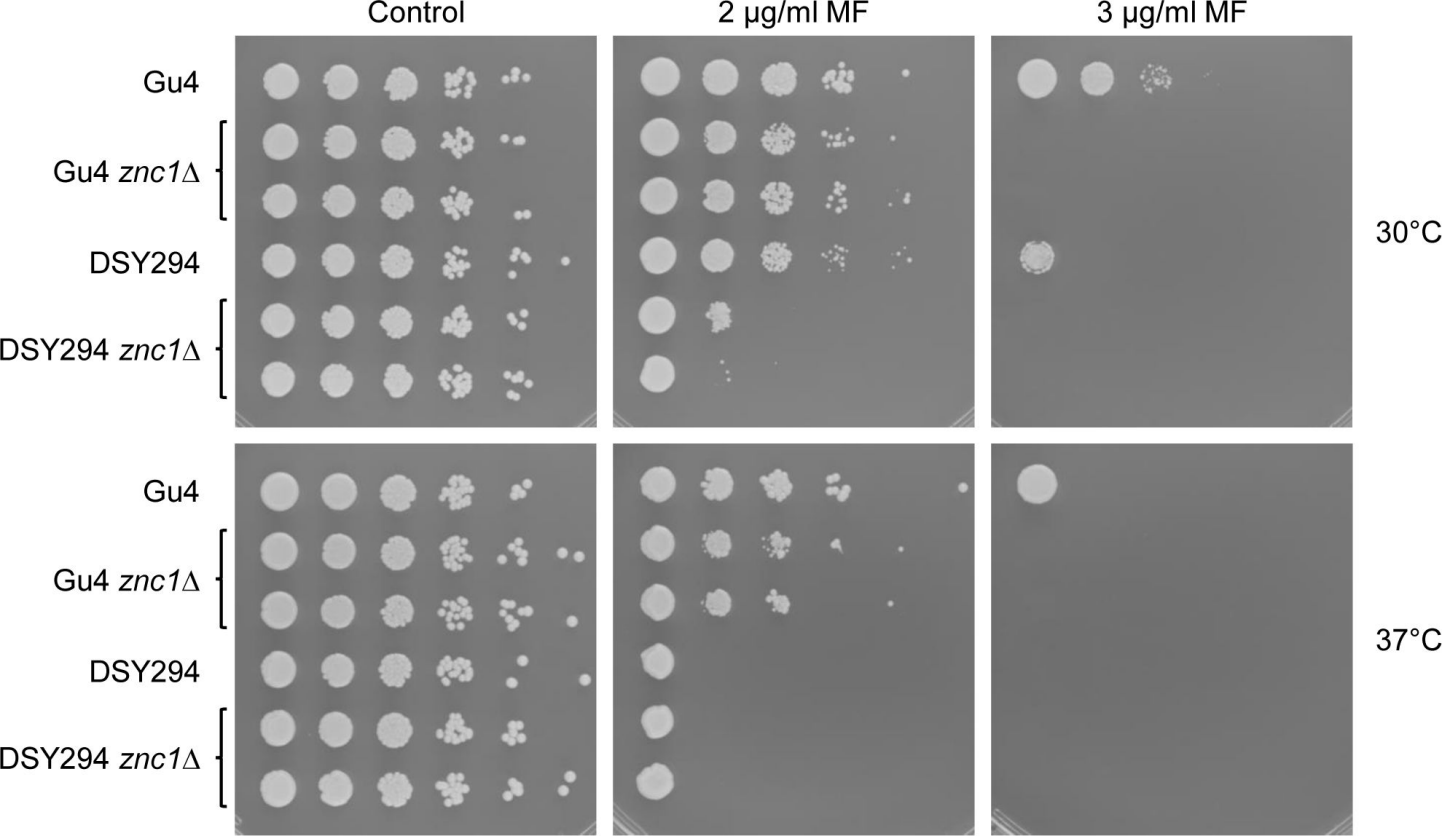
Znc1 is required for miltefosine tolerance in other clinical *C. albicans* isolates. Serial 10-fold dilutions of the clinical isolates Gu4 and DSY294 and two independently generated *znc1*Δ mutants of each parent were spotted on SD agar plates without (control) or with the indicated miltefosine (MF) concentrations and incubated for 2 days at 30°C or 37°C.

The *C. albicans* reference strain SC5314, which was used in our experiments to study Znc1 function, was originally isolated from a patient with disseminated candidiasis (26). To investigate if Znc1 is also required for wild-type miltefosine tolerance in other *C. albicans* strains, we generated *znc1*Δ mutants of two clinical isolates from AIDS patients with oropharyngeal candidiasis, Gu4 and DSY294 (27, 28). Drug sensitivity assays showed that deletion of *ZNC1* in both cases resulted in increased sensitivity to miltefosine (Fig. 5), demonstrating that Znc1 is commonly required for *C. albicans* to tolerate subinhibitory concentrations of this drug.

### Miltefosine resistance in clinical isolates with *TAC1* GOF mutations

**Fig 6:**
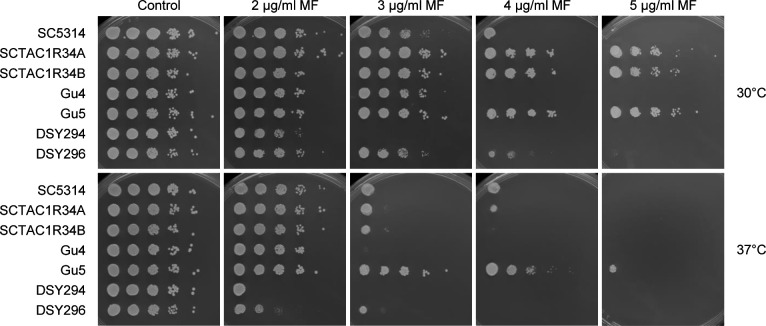
Fluconazole-resistant *C. albicans* isolates with natural *TAC1* GOF mutations display increased miltefosine resistance. Serial 10-fold dilutions of the fluconazole-resistant isolates Gu5 and DSY296 and their matched fluconazole-sensitive isolates Gu4 and DSY294 were spotted on SD agar plates without (control) or with the indicated miltefosine (MF) concentrations and incubated for 2 days at 30°C or 37°C. The reference strain SC5314 and two independently generated derivatives containing the G980E GOF mutation from isolate Gu5 in both *TAC1* alleles were included for comparison.

While no natural GOF mutations in *ZNC1* have been reported so far, *TAC1* GOF mutations are frequently found in fluconazole-resistant *C. albicans* isolates (8–10, 16, 17, 29–31). Although the focus of our present study was Znc1, it was interesting to test if clinical *C. albicans* isolates with hyperactive *TAC1* alleles also exhibited increased miltefosine resistance, similar to the strains with the artificially activated TAC1*. Isolates Gu5 and DSY296 were recovered from later infection episodes of the same patients as isolates Gu4 and DSY294, respectively, but had acquired GOF mutations in *TAC1* that caused *CDR1*/2 overexpression and fluconazole resistance (9, 16). Gu5 and DSY296 also strongly upregulate *RTA3* expression compared with their matched isolates Gu4 and DSY294 with wild-type *TAC1* alleles (4, 18, 19). As shown in Fig. 6, isolates Gu5 and DSY296 were indeed more resistant to miltefosine than the corresponding isolates Gu4 and DSY294, respectively. Furthermore, derivatives of strain SC5314 in which the *TAC1*^G980E^ mutation of isolate Gu5 had been introduced into both endogenous *TAC1* alleles (strains SCTAC1R34A and -B) displayed the same increased miltefosine resistance, albeit only at 30°C and not at 37°C, demonstrating that it was caused by the *TAC1* GOF mutation.

## DISCUSSION

Strains containing constitutively activated forms of zinc cluster transcription factors have proven highly useful to uncover functions of several of these transcription regulators in *C. albicans*, with Tac1 and Mrr1 being prime examples (4, 6). This is also the case for artificially activated ZCFs for which no natural GOF mutations have been described so far. Transcriptional profiling revealed that hyperactive forms of Znc1 and Mrr2 conferred increased fluconazole resistance by upregulating the expression of *CDR1*, encoding the major multidrug efflux pump of *C. albicans*, while an activated form of Stb5 caused upregulation of *YOR1*, encoding another efflux pump, and thereby conferred resistance to oligomycin and beauvericin (3, 32). The finding that a hyperactive Znc1 caused a strong upregulation of *RTA3*, which has been shown to be required for miltefosine tolerance (22), indicated that Znc1 might confer increased resistance to this drug. Indeed, we found that activated forms of Znc1 and Tac1, which cause *RTA3* overexpression, conferred Rta3-dependent increased miltefosine resistance.

Miltefosine-induced *RTA3* upregulation did not depend on Tac1 under our experimental conditions, neither by treatment with 10 µg/mL miltefosine for 60 min (Fig. 3A) nor by treatment with 5 µg/mL miltefosine for 30 min (Fig. 4B). This is in contrast to a previous report by Srivastava et al., in which miltefosine-induced *RTA3* upregulation was found to depend on Tac1 (22). These authors observed only a twofold increase in *RTA3* mRNA levels when cells were treated for 120 min with 5 µg/mL miltefosine, whereas a much stronger induction was found in our experiments. Srivastava et al. (22) also reported an only a twofold increase in *RTA3* expression when cells were treated for 30 min with 10 µg/mL fluphenazine, whereas we and other researchers observed a much stronger induction. Karababa et al. found a >15-fold increase in *RTA3* transcript levels upon treatment of the cells for 20 min with 10 µg/mL fluphenazine (18), and a >30-fold induction of *RTA3* expression after 30 min treatment with 25 µM (~11 µg/mL) fluphenazine was reported by Liu et al. (20). The loss of the mild *RTA3* induction by miltefosine and fluphenazine observed by Srivastava et al. in the absence of Tac1 may be due to the fact that the *tac1*Δ mutant used in their study was an auxotrophic *ura3*Δ strain (DSY2906), which was compared with the wild-type reference strain SC5314 in these experiments. It has been shown previously that the requirement of Tac1 and Znc1 for the upregulation of their common target genes depends on the inducing stimulus. For example, *CDR1* and *RTA3* induction by farnesol could be mediated by either of the two transcription factors, whereas the upregulation of these genes by fluphenazine depended on Tac1 and could not be promoted by Znc1 (20). Our present study demonstrates that Znc1 is the main activator of *RTA3* expression in response to miltefosine, and loss of Znc1 results in hypersensitivity to this drug.

Notably, the increased miltefosine resistance of strains with hyperactive forms of Znc1 and Tac1 was observed within a relatively narrow range of miltefosine concentrations. When grown on SD medium containing 3–4 µg/mL miltefosine, these strains were more resistant than the wild type, but their growth was efficiently inhibited by 5 µg/mL miltefosine (Fig. 1B). This was also reflected in broth microdilution tests, in which only slight or no changes in the minimal inhibitory concentration (MIC) of miltefosine were observed. Even deletion of *RTA3* reduced the MIC only minimally under these conditions (Table S2). The limited increase in miltefosine resistance conferred by hyperactive forms of Znc1 and Tac1 can be explained by the fact that wild-type Znc1 strongly induces *RTA3* expression in the presence of this drug anyway, such that constitutive *RTA3* overexpression confers only a moderate advantage. In *Candida parapsilosis*, amplification of the *RTA3* gene in certain strains has been found to correlate with increased miltefosine resistance (33), similar to the effect of transcriptional upregulation of *RTA3* expression by hyperactive Znc1 and Tac1 in *C. albicans*. Such *RTA3* amplifications were not observed in a large collection of *C. albicans* isolates, and *in vitro* selection for miltefosine resistance did not result in *RTA3* amplification in *C. parapsilosis* but was acquired by inactivating mutations in genes encoding phospholipid translocases that may mediate uptake of miltefosine (a phosphatidylcholine analog) into the cell (33). Whether GOF mutations in Tac1 or Znc1 or other resistance mechanisms confer clinically relevant miltefosine resistance remains to be seen.

## MATERIALS AND METHODS

### Strains and growth conditions

The *C. albicans* strains used in this study are listed in Table S1. All strains were stored as frozen stocks with 17.2% glycerol at −80°C and subcultured on YPD agar plates (10 g yeast extract, 20 g peptone, 20 g glucose, 15 g agar/L) at 30°C. Strains were routinely grown in YPD liquid medium at 30°C in a shaking incubator. For the selection of transformants, 200 µg/mL nourseothricin (Werner Bioagents) was added to YPD agar plates. To obtain nourseothricin-sensitive derivatives in which the *SAT1* flipper cassette was excised by FLP-mediated recombination, transformants were grown overnight in YCB-BSA-YE medium (23.4  g yeast carbon base, 4  g bovine serum albumin, 2  g yeast extract/L, pH 4.0) without selective pressure to induce the *SAP2* promoter controlling *caFLP* expression. Appropriate dilutions were plated on YPD agar plates and grown for 2 days at 30°C. Individual colonies were picked and streaked on YPD plates and on YPD plates with 100  µg/mL nourseothricin to confirm sensitivity.

### Plasmid and strain constructions

An *RTA3* deletion construct was obtained by amplification of the *RTA3* upstream and downstream regions from genomic DNA of strain SC5314 with the primer pairs RTA3.05/RTA3.06 and RTA3.03/RTA3.04, respectively (all oligonucleotide primers used in this study are listed in Table S3). The PCR products were digested with ApaI/XhoI and SacII/SacI, respectively, and cloned on both sides of the *SAT1* flipper cassette contained in plasmid pSFS5 (34) to result in pRTA3M2. To express *RTA3* from the *ADH1* promoter, the *RTA3* coding region (allele 2) was amplified with primers RTA3.01 and RTA3.02. The PCR product was digested with XhoI/BglII and substituted for the *UPC2* coding region of plasmid pUPC2E1 (3), yielding pRTA3E1. *C. albicans* strains were transformed by electroporation (35) with the following gel-purified linear DNA fragments: the insert from pRTA3M2 was used to sequentially delete the *RTA3* wild-type alleles in strain SC5314 and the *cdr1*Δ mutants SCCDR1M4A and SCCDR1M4B (3). The insert from pRTA3E1 was used to express *RTA3* from the *ADH1* promoter in the wild-type strain SC5314 and the *znc1*Δ mutants SCZNC1M4A and -B (3). The insert from pZNC1E1 (3) was used to express *ZNC1* from the *ADH1* promoter in the *znc1*Δ mutants. The cassettes from plasmid pZNC1GAD1 and pTAC1GAD1 (3) were used to introduce the hyperactive ZNC1* and TAC1* alleles into *rta3*Δ single and *cdr1*Δ *rta3*Δ double mutants. The *ZNC1* deletion cassette from plasmid pZNC1M1 (3) was used to generate *znc1*Δ mutants of strains Gu4 and DSY294 as well as *znc1*Δ *tac1*Δ double mutants from *tac1*Δ mutants of strain SC5314 (see Table S1).

### Isolation of genomic DNA and southern hybridization

Genomic DNA from *C. albicans* strains was isolated as described previously (36). The DNA was digested with appropriate restriction enzymes, separated on a 1% agarose gel, transferred by vacuum blotting onto a nylon membrane, and fixed by UV crosslinking. Southern hybridization with enhanced chemiluminescence-labeled probes was performed with the Amersham ECL Direct Nucleic Acid Labelling and Detection System (Cytiva) according to the instructions of the manufacturer.

### Northern hybridization analysis

Overnight cultures of the strains were diluted 10^−2^ in fresh YPD medium and grown for 4 h at 30°C. To detect *RTA3* induction by miltefosine and fluphenazine, 10 µg/mL miltefosine or 10 µg/mL fluphenazine was added after 3 h, and the cultures were incubated for an additional hour. In a separate experiment, the cells were treated for only 30 min with 5 µg/mL miltefosine (added after 3 h 30 min). Total RNA was extracted using a Quick-RNA Fungal/Bacterial Miniprep Kit (Zymo Research) following the manufacturer’s instructions. RNA samples were separated on a 1.2% agarose gel, transferred by capillary blotting onto a nylon membrane, fixed by UV crosslinking, and hybridized with digoxigenin-labeled probes for *RTA3* (positions +139 to +502 in the *RTA3* coding sequence, amplified with primers RTA3_NBF and RTA3_NBR), *ZNC1* (positions +674 to +1018, amplified with primers ZNC1_NBF and ZNC1_NBR), and *ACT1* (positions +1103 to +1591, amplified with primers ACT1NB_F and ACT1NB_R). Bound probe was detected with a peroxidase-labeled anti-digoxigenin AP-conjugate (Roche). Signals were generated using CSPD (Roche) as substrate and captured with the ImageQuant LAS 4000 imaging system (GE Healthcare). Signal intensities were quantified using the image analysis software Fiji (37). Relative *RTA3* expression values reported in the text are the average signal intensities measured in two independently constructed strains using *ACT1* mRNA signals for standardization.

### Susceptibility tests

YPD overnight cultures of the strains were diluted to an optical density at 600 nm of 2.0. Ten-fold serial dilutions were prepared in a 96-well microtiter plate and ca. 5 µL of the cell suspensions was transferred with a replicator onto SD agar plates (0.67% yeast nitrogen base with ammonium sulfate, 2% glucose, 2% agar) without or with various concentrations of miltefosine (Sigma), as indicated in the figures. Miltefosine was dissolved in ethanol; control plates contained the solvent alone. The plates were incubated for 2 days at 30°C or 37°C and photographed. Miltefosine susceptibility was also assayed by a broth microdilution test as follows. A 2-day-old colony from a YPD agar plate was suspended in 2 mL of a 0.9% NaCl solution, and 4 µL of the suspension was mixed with 2 mL 2× SD CSM medium (13.4 g yeast nitrogen base with ammonium sulfate, 40 g glucose, 1.58 g complete supplement medium/L [MP Biomedicals]). A two-fold dilution series of miltefosine was prepared in water, starting from an initial concentration of 32 µg/mL. One hundred microliters of each miltefosine solution was then mixed with 100 µL of the cell suspension in a 96-well microtiter plate, and the plates were incubated at 37°C and visually inspected after 24 h and 48 h. The MIC of miltefosine was defined as the drug concentration that abolished or drastically reduced visible growth compared to that of a drug-free control.

## SUPPLEMENTAL MATERIAL

Fig. S1 msphere.00270-24-s0001.pdf Reintroduction of *ZNC1* under control of the *ADH1* promoter reverts the miltefosine hypersusceptibility of *znc1*Δ mutants.

Table S1 msphere.00270-24-s0002.xlsx *C. albicans* strains used in this study.

Table S2 msphere.00270-24-s0003.xlsx Miltefosine MICs.

Table S3 msphere.00270-24-s0004.xlsx Oligonucleotide primers used in this study.

ASM does not own the copyrights to Supplemental Material that may be linked to, or accessed through, an article. The authors have granted ASM a non-exclusive, world-wide license to publish the Supplemental Material files. Please contact the corresponding author directly for reuse.
