# Supplementary material for: The zinc cluster transcription factor Znc1 regulates Rta3-dependent miltefosine resistance in Candida albicans
Source: mSphere. 2024 Jun 11;9(7):e00270-24. doi: 10.1128/msphere.00270-24 (PMC11288014; doi:10.1128/msphere.00270-24)
Supplement: Fig. S1 — Reintroduction of ZNC1 under control of the ADH1 promoter reverts the miltefosine hypersusceptibility of znc1Δ mutants. [file msphere.00270-24-s0001.pdf]

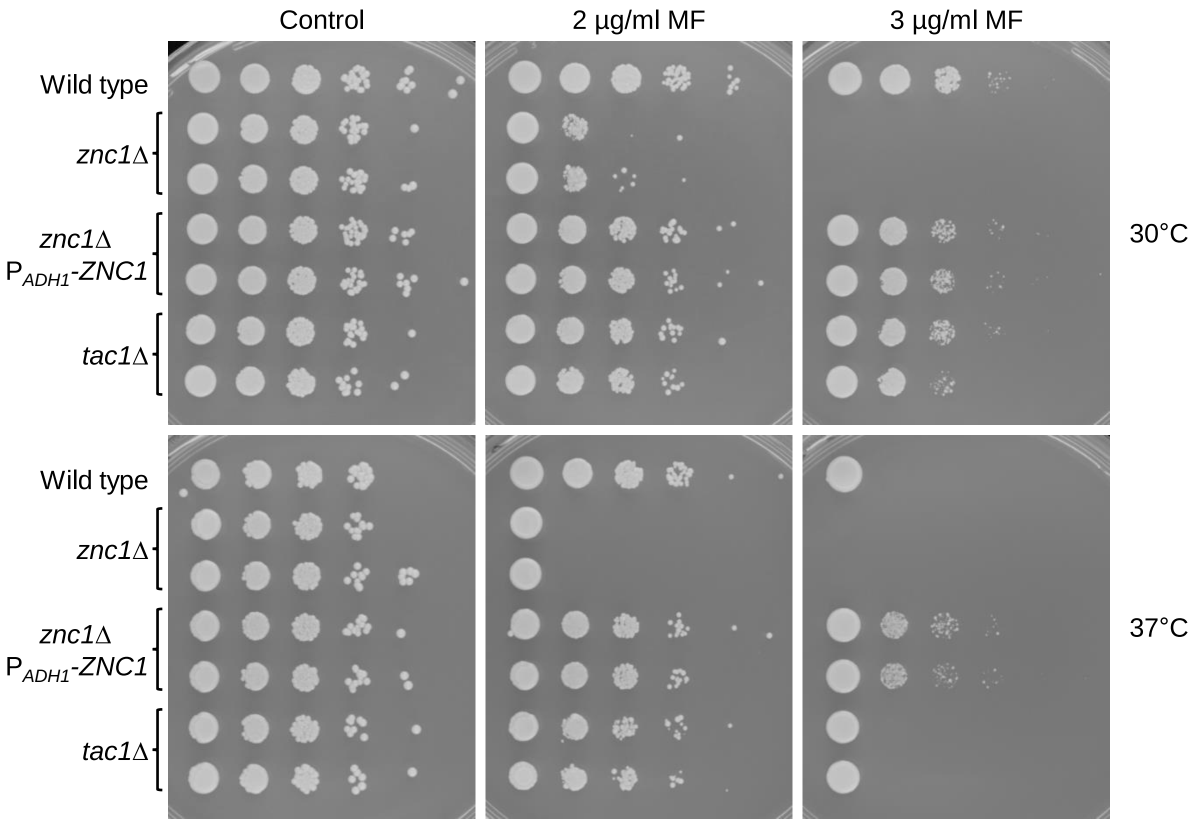

FIG S1 Reintroduction of *ZNC1* under control of the *ADH1* promoter reverts the miltefosine hypersusceptibility of *znc1*Δ mutants. Serial tenfold dilutions of the wild-type strain SC5314, *znc1*Δ mutants and complemented strains, and *tac1*Δ mutants were spotted on SD agar plates without (control) or with the indicated miltefosine (MF) concentrations and incubated for 2 days at 30°C or 37°C.
